# Neural Computing Enhanced Parameter Estimation for Multi-Input and Multi-Output Total Non-Linear Dynamic Models

**DOI:** 10.3390/e22050510

**Published:** 2020-04-30

**Authors:** Longlong Liu, Di Ma, Ahmad Taher Azar, Quanmin Zhu

**Affiliations:** 1 School of Mathematical Sciences, Ocean University of China, Qingdao 266000, China; liulonglong@ouc.edu.cn (L.L.); 21181111013@stu.ouc.edu.cn (D.M.); 2 Robotics and Internet-of-Things Lab (RIOTU), Prince Sultan University, Riyadh 11586, Saudi Arabia; 3 Faculty of Computers and Artificial Intelligence, Benha University, 13511 Benha, Egypt; 4 Department of Engineering Design and Mathematics, University of the West of England, Frenchy Campus Coldharbour Lane, Bristol BS16 1QY, UK; quan.zhu@uwe.ac.uk

**Keywords:** parameter estimation, total non-linear model, neural networks, neuro-computing, gradient descent algorithm

## Abstract

In this paper, a gradient descent algorithm is proposed for the parameter estimation of multi-input and multi-output (MIMO) total non-linear dynamic models. Firstly, the MIMO total non-linear model is mapped to a non-completely connected feedforward neural network, that is, the parameters of the total non-linear model are mapped to the connection weights of the neural network. Then, based on the minimization of network error, a weight-updating algorithm, that is, an estimation algorithm of model parameters, is proposed with the convergence conditions of a non-completely connected feedforward network. In further determining the variables of the model set, a method of model structure detection is proposed for selecting a group of important items from the whole variable candidate set. In order to verify the usefulness of the parameter identification process, we provide a virtual bench test example for the numerical analysis and user-friendly instructions for potential applications.

## 1. Introduction

Because a total non-linear model can provide a very concise representation for complex non-linear systems and has good extrapolation characteristics, it has attracted the attention of academic research and applications. Compared with the polynomial non-linear auto-regressive moving average with exogenous input (NARMAX) model, the total non-linear model is an extension of the polynomial model, which can be defined as the ratio of two polynomial expressions [1,2,3]. The introduction of denominator polynomials makes the NARMAX model non-linear in parameters and regression terms. Therefore, compared with the polynomial model, the model identification and the controller design of the total non-linear model are much more challenging [4,5]. In view of the difficulty of parameter estimation of a total non-linear model, using simple and effective algorithm and machine learning should be considered for extracting the information from measurement data.

### 1.1. Literature Survey

At present, a variety of model structure detection techniques and parameter estimation algorithms are developed for non-linear models, including the orthogonal model structure detection and parameter estimation program [6], the generalized least square estimator [7,8], the prediction error estimator [9,10], the Kalman filter estimator [11,12], the genetic algorithm estimator [12,13], the artificial neural network estimator [14,15,16,17], etc. However, most of these algorithms are parameter estimators for polynomial non-linear models. Zhu and Billings have done a lot of research work on the parameter identification of a total non-linear model [7,8], and they put forward the parameter estimation method of a total non-linear model based on a back-propagation (BP) algorithm in 2003. They discussed the advantages of BP calculation in recognition of the classical model to provide the best combination of classical and neural network methods and provided a powerful tool for analyzing a large number of systems.

In [18], a back-propagation estimation formula based on neuro-computing was presented for estimating the total non-linear model parameters, where a pack of solutions were derived for the problems of parameter initialization, learning rate selection, stop criteria and model structure detection, and the convergence of a back-propagation estimator (BPE). However, Reference [18] only proposed a parameter estimation method for single-input and single-output (SISO) systems, and correspondingly the case studies. Expanding [18], this paper presents solutions for the parameter estimation of a total non-linear multi-input and multi-output (MIMO) model. Due to the complexity of a MIMO system, it is more difficult to estimate the model parameters, but they are more general in academic research and applications. For example, the parameters of a MIMO system are many more than those of a SISO system, and the parameters to be estimated each time will be multiplied, which increases the difficulty of estimation. Moreover, due to the coupling of multiple systems, the parameter values of each system also affect each other. The algorithms to estimate these parameters are not independent but interactive and complex. Because the components of different MIMO systems are different, the total connection neural network structure adopted in [18] is not suitable for estimating the parameters of MIMO systems. When the MIMO system is mapped into a neural network, the network structure is often asymmetric or non-completely connected (the neurons in the hidden layer are not connected with all the neurons in the input layer). That is to say, the network is not a common completely connected feedforward neural network, and the general BP algorithm cannot be directly applied to the estimation of the parameters. Therefore, the learning algorithm of the parameters must be properly derived. Due to the asymmetry of the network, the convergence of the network is also facing challenges. It is necessary to analyze the convergence of the network and give the specific conditions of the network convergence. A MIMO system needs to identify the parameters of a SISO system several times, and a MIMO system can have multiple inputs. In the simulation experiment, the parameters of the system should be estimated under different combinations of multiple inputs, and the performance of the network estimator should be verified. Therefore, the parameter identification of a MIMO system is much more challenging.

### 1.2. Motivation and Contributions

The authors of [19] presented a thorough analysis that included two kernel components, the SISO model and the orthogonal algorithms are parameter estimators for polynomial non-linear models such as predictive and back propagation computation. Since then, rational model identification has gone to diversified directions, such as more theoretical considerations of a non-linear least squares algorithm [4], a maximum likelihood estimation [3], and a biased compensation recursive least squares algorithm [2]. It has been noted that the MIMO rational model identification has seldom attracted research, probably due to the complexity in algorithm formulation and the coupling effect. However, this MIMO rational model identification should be a research agent now because of recent applications and increasing computing facilities.

The total non-linear system model, which is relatively new, is the alternative name of the NARMAX rational model, which was defined by a survey paper on the rational model identification [19]. The total non-linear model emphasizes the non-linearity in both the parameters and control inputs, and it has been taken as a challenging structure for designing non-linear dynamic control systems [1]. The rational model gives more consideration as expanded polynomials in math, structure detection, and parameter estimation in the field of system identification [2,3]. Therefore, the main contribution of the new study is to use neural computing algorithms for a MIMO model parameter estimation. The new study is a complement to those classical NAMAX approaches.

The rest of the paper is organized as follows. The total non-linear model is described in Section 2. Section 3 presents the gradient descent calculation of parameter estimation. Next, model structure detection is discussed in Section 4. A convergence analysis of an algorithm is presented in Section 5. Simulation results and discussions are demonstrated in Section 6. Finally, Section 7 includes the paper conclusions and some of the future aspects.

## 2. Total Non-Linear Model

In mathematics, the dynamic total non-linear model of a MIMO system with error can be defined as

(1) $$y_{i}\left(t\right)={\hat{y}}_{i}+e_{i}\left(t\right)=\frac{a_{i}\left(t\right)}{b_{i}\left(t\right)}+e_{i}\left(t\right)\;=\frac{a_{i}\left(u_{1},u_{2},\ldots ,u_{J},y_{1},y_{2},\ldots ,y_{I},e_{1},e_{2},\ldots ,e_{I}\right)}{b_{i}\left(u_{1},u_{2},\ldots ,u_{J},y_{1},y_{2},\ldots ,y_{I},e_{1},e_{2},\ldots ,e_{I}\right)}+e_{i}\left(t\right)i=1,2,\ldots ,I$$

(2) $$a_{i}\left(t\right)=\sum_{k=1}^{N}p_{k}^{n}\left(t\right)\theta_{k}^{n},b_{i}\left(t\right)=\sum_{k=1}^{D}p_{k}^{d}\left(t\right)\theta_{k}^{d}\;i=1,2,\ldots ,I$$

where $y\left(t\right)=\left[y_{1}\left(t\right),y_{2}\left(t\right),\ldots ,y_{I}\left(t\right)\right]\in R^{I}\;$and $\hat{y}\left(t\right)=\left[{\hat{y}}_{1}\left(t\right),{\hat{y}}_{2}\left(t\right),\ldots ,{\hat{y}}_{I}\left(t\right)\right]\in R^{I}\;$are the measured output and model output, respectively; $u\left(t\right)=\left[u_{1}\left(t\right),u_{2}\left(t\right),\ldots ,u_{J}\left(t\right)\right]\in R^{J}$ is the input; $e\left(t\right)=\left[e_{1}\left(t\right),e_{2}\left(t\right),\ldots ,e_{I}\left(t\right)\right]\in R^{I}$ is the model error; and $t=\left[1,2,\ldots ,T\right]\in Z^{+T}$ is the sampling time index. Numerator$\;a_{i}\left(t\right)\in R$ and denominator $b_{i}\left(t\right)\in R$ as represented by polynomials, regression term $p_{k}^{n}\left(t\right)$, and $p_{k}^{d}\left(t\right)$ are products of past inputs, outputs, and errors, such as $u_{1}\left(t-1\right)y_{2}\left(t-3\right)$*,* $\;u_{1}\left(t-1\right)e_{2}\left(t-2\right)$*,* $y_{2}^{3}\left(t-1\right)$.$\theta^{n}=\left[\theta_{1}^{n},\theta_{2}^{n},\ldots ,\theta_{N}^{n}\right]\in R^{N}$, and $\theta^{d}=\left[\theta_{1}^{d},\theta_{2}^{d},\ldots ,\theta_{D}^{d}\right]\in R^{D}$ are the parameter sets of $a_{i}\left(t\right)$ and $b_{i}\left(t\right)$, respectively.

The task of parameter estimation is to extract the relevant parameter values from the measured input and output data for a given model structure. To form a regression expression for parameter estimation, multiplying $e_{i}\left(t\right)$ of both sides of Formula (1) gives

(3) $$y_{i}\left(t\right)b_{i}\left(t\right)-a_{i}\left(t\right)=b_{i}\left(t\right)e_{i}\left(t\right)$$

**Figure 1:**
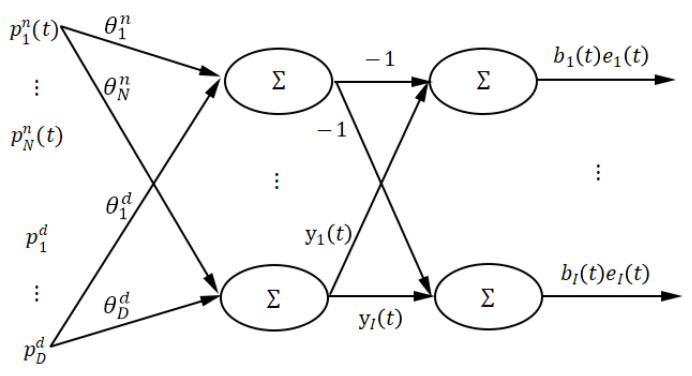
Structure of a neural network corresponding to a total non-linear model.

To consider the neuro-computing approach for parameter estimation, a total non-linear model is expressed into a non-completely connected feedforward neural network, as shown in Figure 1.

We define the network with an on both sides, Formula (11) is obtainedinput layer, a hidden layer, and an output layer, where:

(i) The input layer consists of regression terms $p_{k}^{n}\left(t\right)\left(k=1,\ldots ,N\right)$ and $p_{k}^{d}\left(t\right)\;\left(k=1,\ldots ,D\right)$; here, a neuron in the hidden layer is not connected to all the neurons in the input layer, that is, the network is a non-completely connected feedforward neural network.
(ii) The action function of the neurons in the hidden layer is linear, and the output of the hidden layer neurons is $a_{i}\left(t\right)$ or $b_{i}\left(t\right)$.
(iii) The action function of the output layer neurons is linear, and the output of the *i*th output layer neuron is $b_{i}\left(t\right)e_{i}\left(t\right)$.
(iv) The connection weights between the input layer neurons and the hidden layer neurons are the parameters $\theta_{k}^{n}$ and $\theta_{k}^{d}$ of the model.
(v) The connection weight between the hidden layer neurons and the *i*th output layer neurons are −1 and the observed output $y_{i}\left(t\right)$.

Leung and Haykin proposed a rational function neural network [20] but did not define a generalized total non-linear model structure or consider the relevant errors. Therefore, their parameter estimation algorithm could not provide an unbiased estimation for noise damaged data, which was essentially a special implementation of Zhu and Billings’s [7,8] methods in the case of no noise data. The method proposed in this paper is a further study of the method in Zhu [18]. The characteristics of a total non-linear model (1) are as follows:

(i) By setting parameter i=1, Zhu’s [18] model can be a special case of the model in Formula (1).
(ii) The model is non-linear in parameters and regression terms, which was caused by denominator polynomials.
(iii) When the denominator $b_{i}\left(t\right)$ of the model is close to 0, the output deviation would be large. In this paper, considering this point, division operation was avoided in the action function of the neuron when the neural network model was being built.
(iv) The structure of the neural network corresponding to the total non-linear model is a non-completely connected feedforward neural network, or a partially connected feedforward neural network. Therefore, the convergence of the network becomes a big problem, which is the difficulty of this paper.
(v) The model has a wide range of application prospects. In many non-linear system modeling and control applications, the total non-linear model has been gradually adopted. Some non-linear models, such as the exponential model $e^{x}$, which describes the change of dynamic rate constant with temperature, cannot be directly used. The exponential model can be firstly transformed into a non-linear model ($e^{x}=\frac{1-\frac{x}{2}+\frac{x^{2}}{12}}{1+\frac{x}{2}+\frac{x^{2}}{12}}$), and then, system identification can be implemented [19,21,22].

## 3. Gradient Descent Calculation of Parameter Estimation

For the convenience of the following derivations, set the output of neuron i in the output layer of the neural network as $f_{i}\left(t\right)$.

(4) $$f_{i}\left(t\right)=b_{i}\left(t\right)e_{i}\left(t\right)$$

Define the error measure function of one iteration of network as:

(5) $$E\left(t\right)=\frac{1}{2}\sum^{\text{​}}{\left(y_{i}\left(t\right)-\hat{y_{i}}\left(t\right)\right)}^{2}=\frac{1}{2}\sum^{\text{​}}{\left(e_{i}\left(t\right)\right)}^{2}$$

The Lyapunov method is often used to analyze the stability of a neural network [23]; similarly, the network parameters are estimated by minimizing the network error based on the Lyapunov method. It should be noted that when the total non-linear model is represented in the neural network structure of Figure 1, the parameter estimation of the model can be described as the training of neural network weight by minimizing the error E(t) in Formula (5).

In order to train the weights of the network, the learning algorithm based on the gradient descent is given by Formulas (6) and (7):

(6) $$\Delta \theta_{k}^{n}=-\eta_{n}\frac{\partial E}{\partial \theta_{k}^{n}}\;=-\eta_{n}e_{i}\left(t\right)\frac{\partial e_{i}\left(t\right)}{\partial \theta_{k}^{n}}$$

(7) $$\Delta \theta_{k}^{d}=-\eta_{d}\frac{\partial E}{\partial \theta_{k}^{d}}\;=-\eta_{d}e_{i}\left(t\right)\frac{\partial e_{i}\left(t\right)}{\partial \theta_{k}^{d}}$$

where $\eta_{n}$ and $\eta_{d}$ are learning rates.

By deriving Formula (4) from $\theta_{k}^{n}$ on both sides, Formula (8) is obtained:

(8) $$\frac{\partial f_{i}\left(t\right)}{\partial \theta_{k}^{n}}=\frac{\partial b_{i}\left(t\right)}{\partial \theta_{k}^{n}}e_{i}\left(t\right)\;+b_{i}\left(t\right)\frac{\partial e_{i}\left(t\right)}{\partial \theta_{k}^{n}}\;\frac{\partial e_{i}\left(t\right)}{\partial \theta_{k}^{n}}\;=\frac{1}{b_{i}\left(t\right)}\left(\frac{\partial f_{i}\left(t\right)}{\partial \theta_{k}^{n}}-\frac{\partial b_{i}\left(t\right)}{\partial \theta_{k}^{n}}e_{i}\left(t\right)\right)=\frac{1}{b_{i}\left(t\right)}\frac{\partial f_{i}\left(t\right)}{\partial \theta_{k}^{n}}=\frac{1}{b_{i}\left(t\right)}\frac{\partial \left(y_{i}\left(t\right)b_{i}\left(t\right)-a_{i}\left(t\right)\right)}{\partial \theta_{k}^{n}}=-\frac{1}{b_{i}\left(t\right)}\frac{\partial a_{i}\left(t\right)}{\partial \theta_{k}^{n}}=-\frac{p_{k}^{n}\left(t\right)}{b_{i}\left(t\right)}$$

Substituting Formula (8) into Formula (6) to get Formula (9), we can then get Formula (10):

(9) $$\Delta \theta_{k}^{n}=-\eta_{n}e_{i}\left(t\right)\frac{\partial e_{i}\left(t\right)}{\partial \theta_{k}^{n}}=\eta_{n}e_{i}\left(t\right)\frac{p_{k}^{n}\left(t\right)}{b_{i}\left(t\right)}$$

(10) $$\theta_{k}^{n}\left(t+1\right)=\theta_{k}^{n}\left(t\right)+\Delta \theta_{k}^{n}=\theta_{k}^{n}\left(t\right)+\eta_{n}e_{i}\left(t\right)\frac{p_{k}^{n}\left(t\right)}{b_{i}\left(t\right)}$$

By deriving Formula (4) from $\theta_{k}^{d}$ on both sides, Formula (11) is obtained:

(11) $$\frac{\partial f_{i}\left(t\right)}{\partial \theta_{k}^{d}}=\frac{\partial b_{i}\left(t\right)}{\partial \theta_{k}^{d}}e_{i}\left(t\right)\;+b_{i}\left(t\right)\frac{\partial e_{i}\left(t\right)}{\partial \theta_{k}^{d}}\;\frac{\partial e_{i}\left(t\right)}{\partial \theta_{k}^{d}}\;=\frac{1}{b_{i}\left(t\right)}\left(\frac{\partial f_{i}\left(t\right)}{\partial \theta_{k}^{d}}-\frac{\partial b_{i}\left(t\right)}{\partial \theta_{k}^{d}}e_{i}\left(t\right)\right)\;=\frac{1}{b_{i}\left(t\right)}\left(\frac{\partial \left(y_{i}\left(t\right)b_{i}\left(t\right)-a_{i}\left(t\right)\right)}{\partial \theta_{k}^{d}}-\frac{\partial b_{i}\left(t\right)}{\partial \theta_{k}^{d}}e_{i}\left(t\right)\right)=\frac{1}{b_{i}\left(t\right)}\left(y_{i}\left(t\right)p_{k}^{d}\left(t\right)-p_{k}^{d}\left(t\right)e_{i}\left(t\right)\right)\;=\frac{1}{b_{i}\left(t\right)}\left(y_{i}\left(t\right)-e_{i}\left(t\right)\right)p_{k}^{d}\left(t\right)=\frac{1}{b_{i}\left(t\right)}\;\frac{a_{i}\left(t\right)}{b_{i}\left(t\right)}p_{k}^{d}\left(t\right)=\frac{a_{i}\left(t\right)}{b_{i}^{2}\left(t\right)}p_{k}^{d}\left(t\right)$$

Substituting Formula (11) into Formula (8) to get Formula (12), we then get Formula (13):

(12) $$\Delta \theta_{k}^{d}=-\eta_{d}e_{i}\left(t\right)\frac{\partial e_{i}\left(t\right)}{\partial \theta_{k}^{d}}=-\eta_{d}e_{i}\left(t\right)\frac{a_{i}\left(t\right)}{b_{i}^{2}\left(t\right)}p_{k}^{d}\left(t\right)$$

(13) $$\theta_{k}^{d}\left(t+1\right)=\theta_{k}^{d}\left(t\right)+\Delta \theta_{k}^{d}\;=\theta_{k}^{d}\left(t\right)-\eta_{d}e_{i}\left(t\right)\frac{a_{i}\left(t\right)}{b_{i}^{2}\left(t\right)}p_{k}^{d}\left(t\right)$$

The gradient descent algorithm for parameter estimation of a total non-linear model is summarized in Algorithm 1.
**Algorithm 1.** Gradient Descent Algorithm 1: Initialization: The weights of the neural network (parameters of a total non-linear model) are set as random little numbers with uniform distribution; the average value is zero, and the variance is small. Set the maximum number of iterations T, the minimum error ε, and the maximum number of samples P. 2: Generate training sample set {X, Y} of the neural network according to Formula (1), where $X=\left\{X_{1},X_{2},\ldots ,X_{I}\right\}$, Y = {$Y_{1},Y_{2},\ldots ,Y_{I}$},   $X_{i}∋${$p_{1}^{n}\left(t\right)$,$\;p_{2}^{n}\left(t\right)$,…,$\;p_{N}^{n}\left(t\right),\;p_{1}^{d}$,$p_{2}^{d},\ldots ,p_{D}^{d}$}, $Y_{i}$={$y_{i}\left(t\right)$}. 3: Input a training sample *p* to the neural network. 4: Calculate the output value $a_{i}(k)$,$y_{i}(t)e_{i}(t)$ and $f_{i}\left(t\right)$ of the neurons in the hidden layer and the output layer according to Formulas (2), (3), and (4), respectively. 5: Adjust the weight of the neural network according to Formulas (10) and (13). 6: Calculate the error E(t) according to Formula (4) and calculate the total error according to Formula (14).(14)$$E=\sum^{\text{​}}E\left(t\right)$$ 7: p=p+1 8: If *p* > *P*, then t = t + 1; otherwise, run step 3. 9: If E< ε or t>T, stop training; otherwise, run step 3.

## 4. Model Structure Detection

Model structure detection is to select important items from a rather large model set (usually called the whole item set) and determine the sub-model with important items [18]. Because of the powerful self-learning and associative memory function of an artificial neural network [24], it is the first-choice tool to identify the model structure. When identifying systems with unknown structures, it is important to avoid losing these important items in the final model. For the structure detection of a total non-linear model, the connection weight estimation in the neural network, that is, the parameter estimation of the total non-linear model, could be used to select the significant terms.

For the important and unimportant items in the whole model item set, the knock-out algorithm is adopted. First, remove the items that lead to the increase of network error, and then knock out the items with lighter weight according to the requirements of significance. Finally, test the error of the non-linear model composed of the remaining items. The specific algorithm is summarized in Algorithm 2.
**Algorithm 2.** Knock-Out Algorithm1: Using the network structure shown in Figure 1, all the items contained in the whole items set are taken as the input of the network. 2: The algorithm in Section 3 is used to train the network, and network error $E_{1}$ is obtained. 3: A new network structure is obtained by randomly removing a network input. The algorithm in Section 3 is used to train the new network, and network error $E_{2}$ is obtained. If $E_{2}\leq E_{1}$, then $E_{1}=E_{2}$. Otherwise, this operation should be invalid (the input is reserved). 4: Another input is selected, and step 3 is executed again until all the input items are executed once. 5: The *N* connection weights between the input layer and the hidden layer are sorted in descending order. The first *n* weights are selected to make the significance reach 95%. Meanwhile, Formulas (15) and (16) are met, and the network input items corresponding to the first *n* weights are retained.(15)$$\frac{\sum_{i=1}^{n}\left|w_{i}\right|}{\sum_{i=1}^{N}\left|w_{i}\right|}\geq 0.95$$
(16)$$\frac{\sum_{i=1}^{n-1}\left|w_{i}\right|}{\sum_{i=1}^{N}\left|w_{i}\right|}<0.95$$

In the above process, the neural network is not only used to estimate the parameters of the model but also to detect the structure of the model and analyze the significance of the regression term.

## 5. Convergence Analysis of the Algorithm

Convergence proof:

Assuming that a connection weight of the neural network shown in Figure 1 is changed, this weight can take any value. When the weight $\theta_{k}^{n}\;$corresponds to the regression term parameter of the numerator of the total non-linear model, the resulting network error changes as follows (remove the lower corner marks in Formula (2) for the convenience of proof):

(17) $$y\left(t\right)=\frac{a\left(t\right)}{b\left(t\right)}+e\left(t\right)$$

Substitute Formula (2) into Formula (1) to get Formula (18):

(18) $$y\left(t\right)-\frac{\sum_{n=1}^{N}p_{k}^{n}\left(t\right)\theta_{k}^{n}}{b\left(t\right)}=e\left(t\right)$$

When $\theta_{k}^{n}$ is updated, (18) becomes (19):

(19) $$y\left(t\right)-\frac{\sum_{n=1,n\neq j}^{N}p_{k}^{n}\left(t\right)\theta_{k}^{n}+p_{k}^{n}\left(t\right)\left(\theta_{k}^{n}+\Delta \theta_{k}^{n}\right)}{b\left(t\right)}=\tilde{e}\left(t\right)$$

$\tilde{e}\left(t\right)$ is the new error of the neural network after the weight has been updated. Subtract Formula (18) from Formula (19) to get Formulas (20) and (21):

(20) $$\tilde{e}\left(t\right)-e\left(t\right)=-\frac{p_{k}^{n}\left(t\right)\Delta \theta_{k}^{n}}{b\left(t\right)}\;=-\eta_{n}{\left(\frac{p_{k}^{n}\left(t\right)}{b\left(t\right)}\right)}^{2}\;e\left(t\right)\;\tilde{e}\left(t\right)\;=(1-\eta_{n}\left(\frac{p_{k}^{n}\left(t\right)}{b\left(t\right)}{)}^{2}\right)\;e\left(t\right)$$

(21) $$\tilde{e}{\left(t\right)}^{2}=(1-\eta_{n}\left(\frac{p_{k}^{n}\left(t\right)}{b\left(t\right)}{)}^{2}\right){}^{2}e{\left(t\right)}^{2}$$

In order to ensure $\tilde{e}{\left(t\right)}^{2}\leq e{\left(t\right)}^{2}$, $-1\leq 1-\eta_{n}{\left(\frac{p_{k}^{n}\left(t\right)}{b\left(t\right)}\right)}^{2}\leq 1$, namely:

(22) $$\{\begin{array}{c} \eta_{n}{\left(\frac{p_{k}^{n}\left(t\right)}{b\left(t\right)}\right)}^{2}\leq 2\\ \eta_{n}{\left(\frac{p_{k}^{n}\left(t\right)}{b\left(t\right)}\right)}^{2}\geq 0 \end{array}$$

Solving Formula (22) gives:

(23) $$0\leq \eta_{n}\leq \frac{2b{\left(t\right)}^{2}}{p_{k}^{n}{\left(t\right)}^{2}}$$

When the changed weight $\theta_{k}^{d}$ corresponds to the regression parameter of the denominator of the total non-linear model, the resulting network error change is as follows:

(24) $$y\left(t\right)-\frac{a\left(t\right)}{\sum_{d=1}^{D}p_{k}^{d}\left(t\right)\theta_{k}^{d}}=e\left(t\right)$$

(25) $$y\left(t\right)-\frac{a\left(t\right)}{\sum_{d=1,d\neq j}^{D}p_{k}^{d}\left(t\right)+p_{k}^{d}\left(t\right)\left(\theta_{k}^{d}+\Delta \theta_{k}^{d}\right)}=\tilde{e}\left(t\right)$$

Subtracting Formula (24) from Formula (25) gives $\tilde{b}\left(t\right)$ as the new denominator of the neural network after the weight has been updated.

(26) $$\tilde{e}\left(t\right)-e\left(t\right)=\frac{\left(\tilde{b}\left(t\right)-b\left(t\right)\right)a\left(t\right)}{\tilde{b}\left(t\right)b\left(t\right)}=p_{k}^{d}\left(t\right)\Delta \theta_{k}^{d}\frac{a\left(t\right)}{\tilde{b}\left(t\right)b\left(t\right)}=-\eta_{d}e\left(t\right)\frac{\partial e\left(t\right)}{\partial \theta_{k}^{d}}p_{k}^{d}\left(t\right)\frac{a\left(t\right)}{\tilde{b}\left(t\right)b\left(t\right)}\;=-\eta_{d}e\left(t\right)\frac{a{\left(t\right)}^{2}}{\tilde{b}\left(t\right)b{\left(t\right)}^{3}}p_{k}^{d}{\left(t\right)}^{2}$$

(27) $$\tilde{e}{\left(t\right)}^{2\;}={\left(1-\eta_{d}\frac{a{\left(t\right)}^{2}}{\tilde{b}\left(t\right)b{\left(t\right)}^{3}}p_{k}^{d}{\left(t\right)}^{2}\right)}^{2}\;e{\left(t\right)}^{2}$$

In order to satisfy $\tilde{e}{\left(t\right)}^{2}\leq e{\left(t\right)}^{2}$, namely, $-1\leq 1-\eta_{d}\frac{a{\left(t\right)}^{2}}{\tilde{b}\left(t\right)b{\left(t\right)}^{3}}p_{k}^{d}{\left(t\right)}^{2}\leq 1$, that is:

(28) $$\{\begin{array}{c} \eta_{d}\frac{a{\left(t\right)}^{2}}{\tilde{b}\left(t\right)b{\left(t\right)}^{3}}p_{k}^{d}{\left(t\right)}^{2}\leq 2\\ \eta_{d}\frac{a{\left(t\right)}^{2}}{\tilde{b}\left(t\right)b{\left(t\right)}^{3}}p_{k}^{d}{\left(t\right)}^{2}\geq 0 \end{array}$$

Because the learning coefficient is too large, the training effect of the network is not effective; accordingly, we take $0\leq \eta_{n}\leq 1$ to get $\tilde{b}\left(t\right)b\left(t\right)>0$, and thus, it has:

(29) $$0\leq \eta_{d}\leq \frac{2\tilde{b}\left(t\right)b{\left(t\right)}^{3}}{a{\left(t\right)}^{2}p_{k}^{d}{\left(t\right)}^{2}}$$

To sum up, the network is convergent when the following conditions are met:

$$1.\;0\leq \eta_{n}\leq \frac{2b{\left(t\right)}^{2}}{p_{k}^{n}{\left(t\right)}^{2}}$$

$$2.\;0\leq \eta_{d}\leq \frac{2\tilde{b}\left(t\right)b{\left(t\right)}^{3}}{a{\left(t\right)}^{2}p_{k}^{d}{\left(t\right)}^{2}}$$

Under these two conditions, this algorithm provides a convergence estimate for the parameters of the total non-linear model.

## 6. Simulation Results and Discussions

Consider a representative example of a total non-linear model:

(30) $$y_{1}\left(t\right)\;=\frac{0.5y_{1}\left(t-1\right)+0.8y_{2}^{3}\left(t-2\right)+u_{1}\left(t-1\right)}{1+y_{1}^{2}\left(t-1\right)+u_{2}^{2}\left(t-1\right)}+r_{1}\left(t\right)\;=\frac{\theta_{1}y_{1}\left(t-1\right)+\theta_{2}y_{2}^{3}\left(t-2\right)+\theta_{3}u_{1}\left(t-1\right)}{1+\theta_{4}y_{1}^{2}\left(t-1\right)+\theta_{5}u_{2}^{2}\left(t-1\right)}+r_{1}\left(t\right)$$

(31) $$y_{2}\left(t\right)\;=\frac{0.2y_{2}\left(t-1\right)-0.5y_{1}^{2}\left(t-2\right)+u_{2}\left(t-1\right)}{1+y_{2}^{2}\left(t-1\right)+u_{2}^{2}\left(t-1\right)}+r_{2}\left(t\right)\;=\frac{\theta_{6}y_{2}\left(t-1\right)-\theta_{7}y_{1}^{2}\left(t-2\right)+\theta_{8}u_{2}\left(t-1\right)}{1+\theta_{9}y_{2}^{2}\left(t-1\right)+\theta_{10}u_{2}^{2}\left(t-1\right)}+r_{2}\left(t\right)$$

Because the disturbance of input data will cause interference to the estimation of parameters [25], in this section, the parameter estimation for different inputs was selected. Firstly, for the simulation system without noise, 2000 pairs of input/output data were used as data sets for uniform sampling in 20 cycles, and the learning rate was designed as a linear attenuation sequence (in 50 iterations, the learning rate decreases from η_0_ = 0.5 to η_end_ = 0.02). The algorithm in this paper was used to estimate 10 parameters at the same time. Table 1, after 50 iterations, shows the estimated values and mean square deviation of parameters.

**Figure 2:**
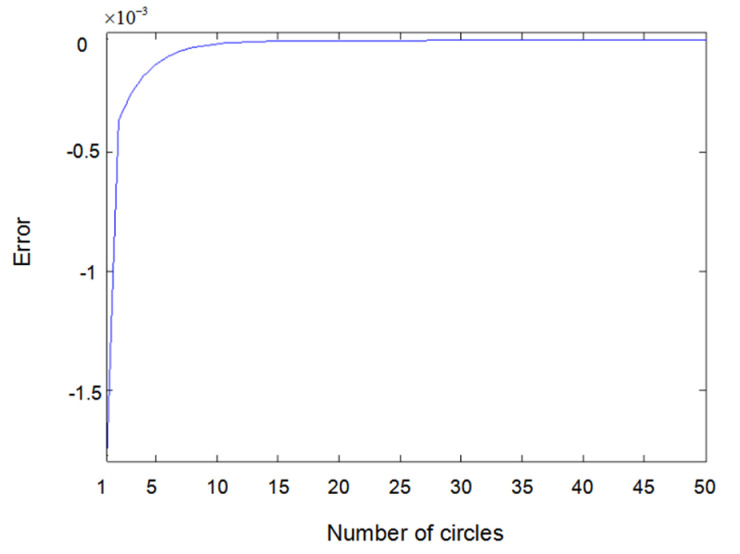
Error of $y_{1}\left(t\right)$ with sine–sine input.

**Figure 3:**
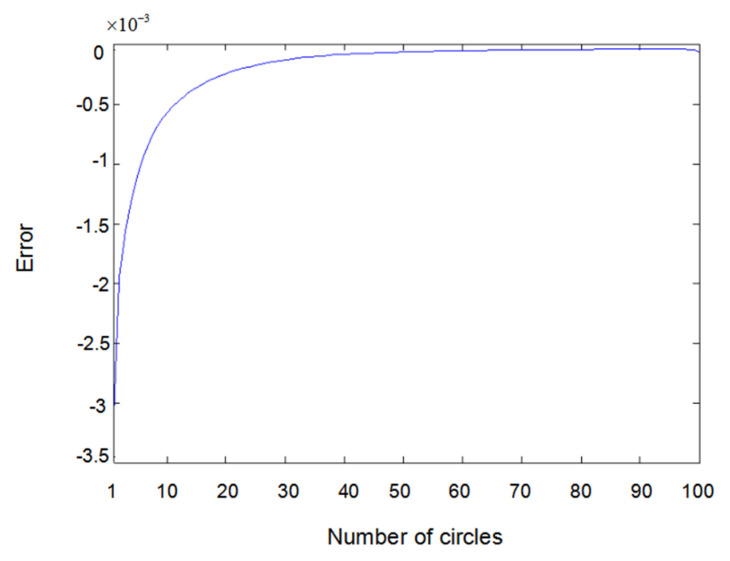
Error of $y_{2}\left(t\right)$ with sine–sine input.

**Figure 4:**
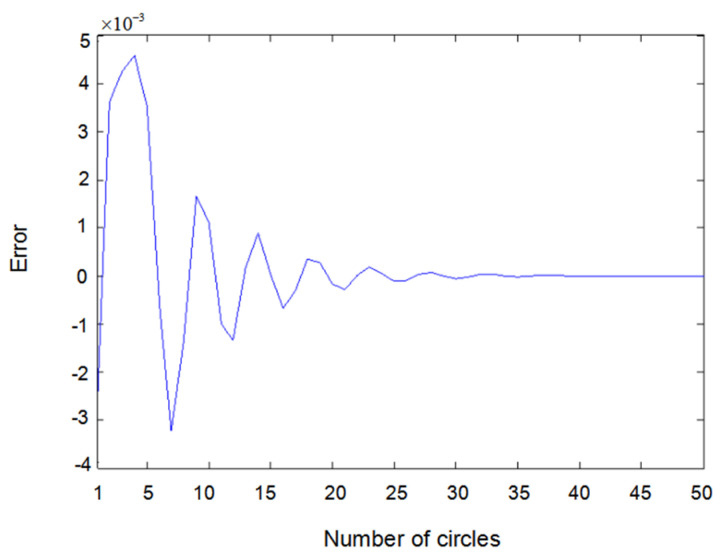
Error of $y_{1}\left(t\right)$ with sine–square input.

The inputs $u_{1}\left(t\right)$ and $u_{2}\left(t\right)$ of the system are either a sine wave or square wave with an amplitude of 2. Figure 2 shows the difference between the measured value $y_{1}\left(t\right)$ (the real output value of Formula (6.1)) and the output value ${\hat{y}}_{1}\left(t\right)\;$obtained using the parameter estimator when inputs $u_{1}\left(t\right)$ and $u_{2}\left(t\right)$ are both sine waves. In the same way, Figure 3 shows the difference between measured value $y_{2}\left(t\right)$ and output value ${\hat{y}}_{2}\left(t\right)$ when inputs $u_{1}\left(t\right)$ and $u_{2}\left(t\right)$ are both sine waves. Figure 4 shows the difference between measured value $y_{1}\left(t\right)$ and output value ${\hat{y}}_{1}\left(t\right)$ when input $u_{1}\left(t\right)$ is a sine wave and $u_{2}\left(t\right)$ is a square wave.

**Figure 5:**
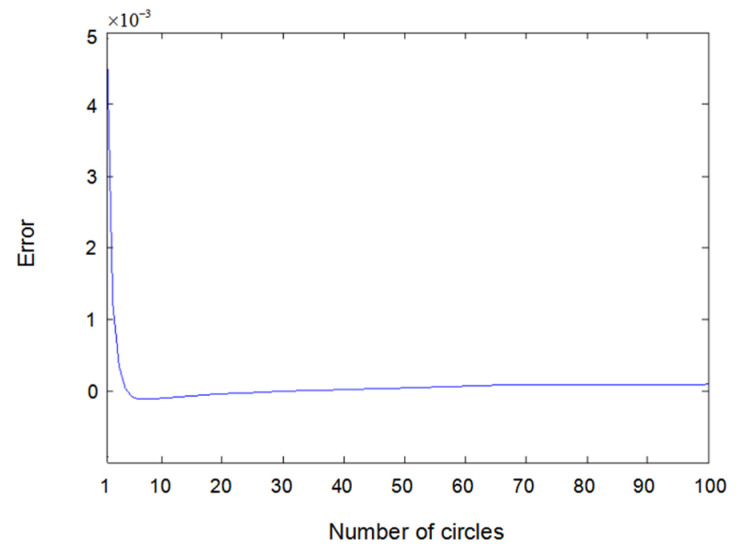
Error of $y_{2}\left(t\right)$ with sine–square input.

**Figure 6:**
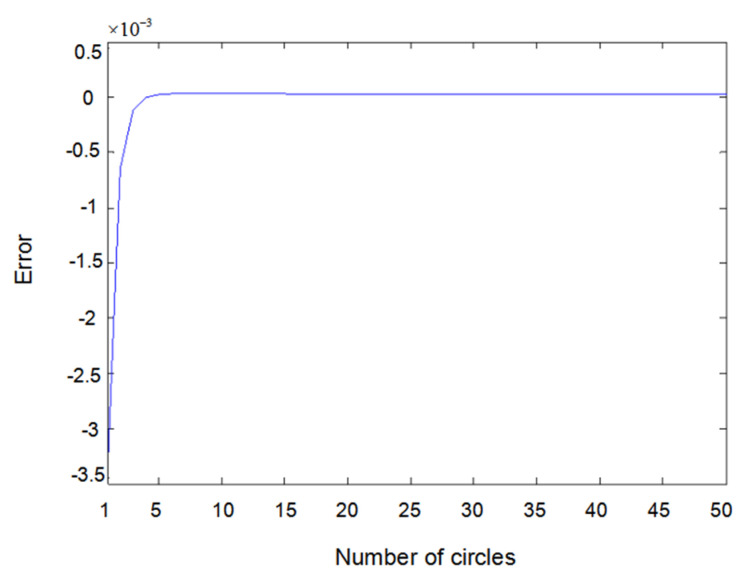
Error of $y_{1}\left(t\right)$ with square–square input.

**Figure 7:**
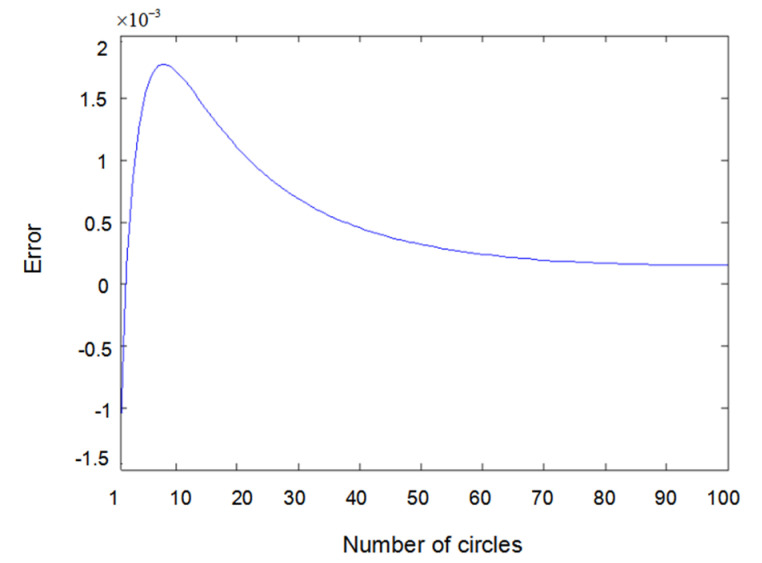
Error of $y_{2}\left(t\right)$ with square–square input.

Figure 5 shows the difference between measured value $y_{2}\left(t\right)$ and output value ${\hat{y}}_{2}\left(t\right)$ when input $u_{1}\left(t\right)$ is a sine wave and $u_{2}\left(t\right)$ is square wave. Figure 6 shows the difference between measured value $y_{1}\left(t\right)$ and output value ${\hat{y}}_{1}\left(t\right)$ when input $u_{1}\left(t\right)$ and $u_{2}\left(t\right)$ are both square waves. Figure 7 shows the difference between measured value $y_{2}\left(t\right)$ and output value ${\hat{y}}_{2}\left(t\right)$ when input $u_{1}\left(t\right)$ and $u_{2}\left(t\right)$ are both square waves. It can be seen that when the inputs are both sine waves, the accuracy of parameter estimation is the highest, while when the inputs are square waves, the estimation accuracy of the parameters is relatively lower. This is because when the inputs are square waves, the output of the system has an overshoot, that is to say, the observation value of the system itself has an error. Training the network with error data will certainly lead to an error of parameter estimation; especially the estimation error of $\theta_{2}$ is the highest. Here, $\theta_{2}$ is the parameter of $y_{2}^{3}\left(t-2\right)$ because $y_{2}^{3}\left(t-2\right)$ is the third power of the output, which further amplifies the error. Substituting the value of $y_{2}^{3}\left(t-2\right)$ into the update of $\theta_{2}$ would inevitably lead to an estimation error.

**Figure 8:**
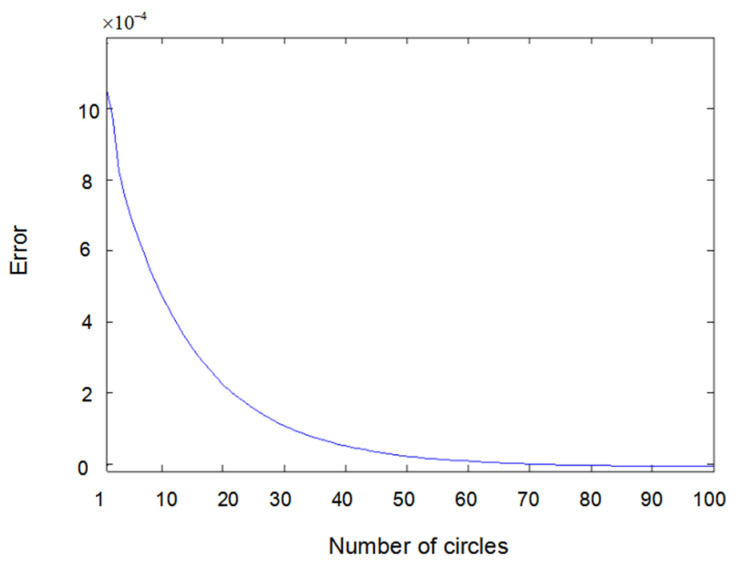
Error of $y_{1}\left(t\right)$ with noise and sine–sine input.

**Figure 9:**
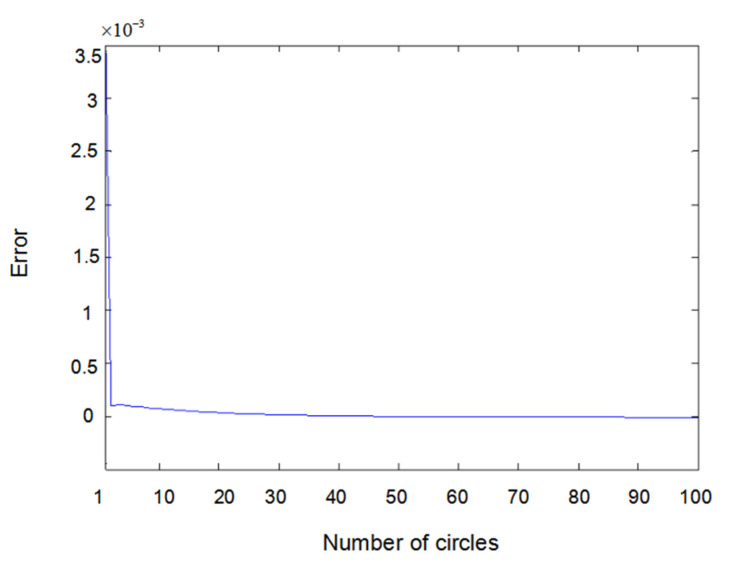
Error of $y_{2}\left(t\right)$ with noise and sine–sine input.

**Figure 10:**
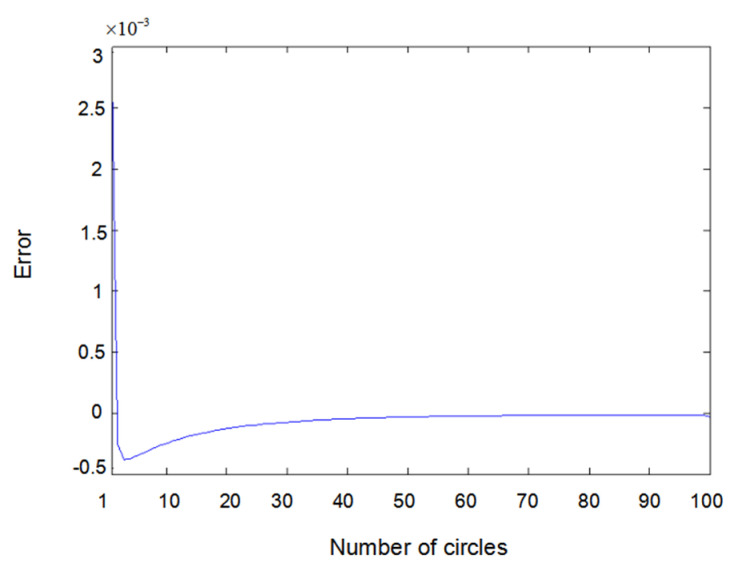
Error of $y_{1}\left(t\right)$ with noise and sine–square input.

**Figure 11:**
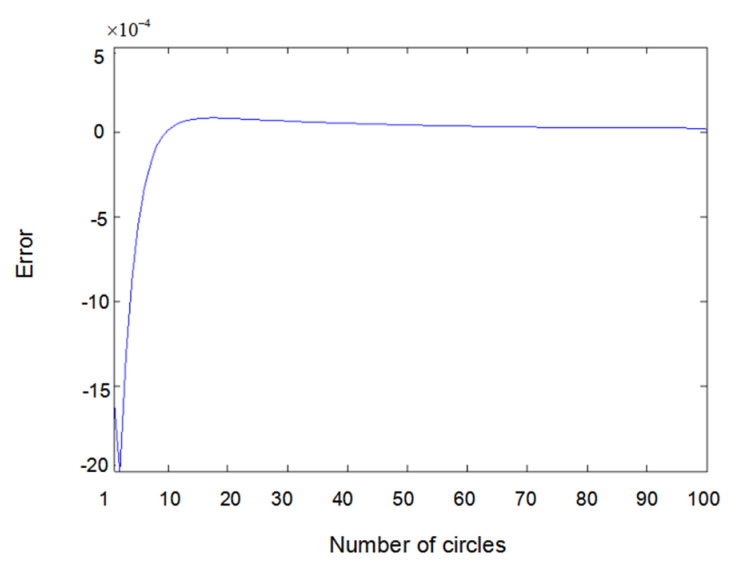
Error of $y_{2}\left(t\right)$ with noise and sine–square input.

**Figure 12:**
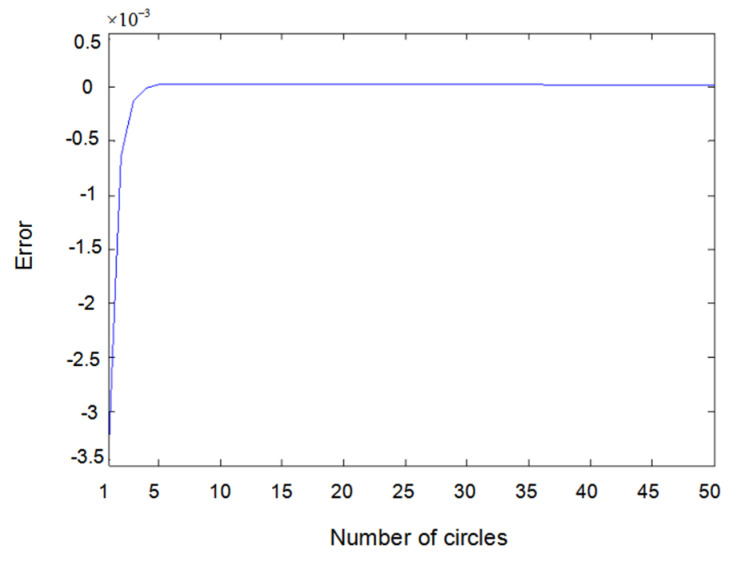
Error of $y_{1}\left(t\right)$ with noise and square–square input.

**Figure 13:**
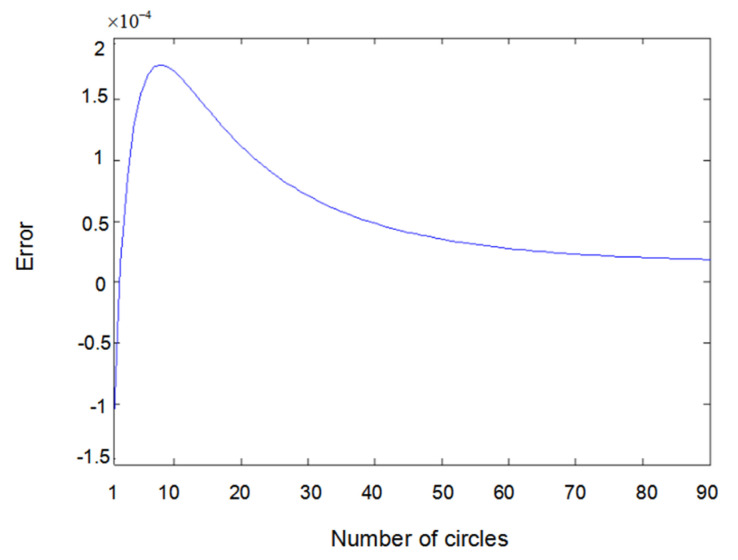
Error of $y_{2}\left(t\right)\;$with noise and square–square input.

For a system with noise interference, it is more difficult to estimate its parameters because of the error of the measured value itself [26,27]. In order to further verify the performance of the estimator, a system with noise was selected for parameter estimation, that is, by adding a noise signal to the original system. The noises $r_{1}\left(t\right)$ and $r_{2}\left(t\right)$ were random uniform sequences, where each mean value was zero, each variance was 0.1, and each signal-to-noise ratio was 10. We repeated the previous experiment for the system with noise, and the experimental results are shown in Table 2. The difference between the measured value and the estimated value of the system output is shown in Figure 8, Figure 9, Figure 10, Figure 11, Figure 12 and Figure 13.

In order to detect the structure of the model, 20 items including 10 items in Formula (6.1) were used as the whole items set of models. The newly added numerator term is of order 1, the denominator term is of order 2, and the input lag and output lag are both of order 1. Using the knock-out algorithm in Section 4, the final 10 items of the model are in good agreement with those in Formula (6.1).

From the above experimental results, the estimation accuracy of the algorithm proposed in this paper is acceptable, and the mean square deviations are all less than 0.003. This level of error is acceptable.

## 7. Conclusions

In this paper, the parameter estimation of a SISO rational model was extended to that of a MIMO total non-linear model. A method of parameter estimation of a MIMO non-linear rational model based on a gradient descent algorithm was proposed, and the convergence condition was proposed for the asymmetry of the network. It was proven that the estimator is properly effective by mathematical derivation and simulation. This estimation method has a strong generalization property and could be widely used in many fields, such as non-linear system modeling and control applications. Some systems that could not directly use this method, such as the exponential model describing the change of the kinetic rate constant with the temperature, could first be converted into a rational model and then use the developed estimation method. Some of the future work could be foreseen as (1) estimating the parameters of the state space model based on an artificial neural network, (2) estimating the parameters of a MIMO state space model, (3) estimating the parameters of the non-linear state space model, and (4) estimating the parameters of total non-linear spatial state models.

## Figures and Tables

**Table 1:** Parameter estimation of a noiseless system.

$u_{1}\left(t\right)$	$u_{2}\left(t\right)$	$\theta_{1}$	$\theta_{2}$	$\theta_{3}\;$	$\theta_{4}\;$	$\theta_{5}\;$	$\theta_{6}\;$	$\theta_{7}\;$	$\theta_{8}\;$	$\theta_{9}\;$	$\theta_{10}\;$	MSE
**sine**	**sine**	0.5002	0.8025	1.0003	1.0034	1.0000	0.2006	0.5010	1.0004	1.0018	0.9991	2.351E-06
**sine**	**square**	0.5000	0.8000	1.0000	1.0000	1.0000	0.1996	0.4982	1.0182	0.9677	1.0473	0.0003
**square**	**square**	0.4973	0.8760	1.0110	1.0031	1.0153	0.2013	0.5072	1.0354	0.9744	1.0840	0.0015

**Table 2:** Parameter estimation of a noisy system.

$u_{1}\left(t\right)$	$u_{2}\left(t\right)$	$\theta_{1}$	$\theta_{2}$	$\theta_{3}\;$	$\theta_{4}\;$	$\theta_{5}\;$	$\theta_{6}\;$	$\theta_{7}\;$	$\theta_{8}\;$	$\theta_{9}\;$	$\theta_{10}\;$	*MSE*
**sine**	**sine**	0.5003	0.8041	1.0005	1.0054	1.0001	0.2008	0.5014	1.0005	1.0016	0.9987	5.342E-06
**sine**	**square**	0.5000	0.8001	1.0000	1.0001	1.0000	0.2045	0.5019	1.073	1.1364	1.0898	0.0032
**square**	**square**	0.4953	0.8765	1.0085	1.0327	1.0095	0.2969	0.7030	0.9971	1.0007	0.9953	0.0058

## References

[1] Billings S.A., Chen S. (1989). Identification of non-linear rational systems using a prediction-error estimation algorithm. Int. J. Syst. Sci..

[2] Billings S.A., Zhu Q.M. (1991). Rational model identification using an extended least-squares algorithm. Int. J. Control.

[3] Sontag E.D. (1979). Polynomial Response Maps. Lecture Notes in Control & Information Sciences.

[4] Narendra K.S., Parthasarathy K. (2002). Identification and control of dynamical systems using neural networks. IEEE Trans. Neural Netw..

[5] Zhu Q.M., Ma Z., Warwick K. (1999). Neural network enhanced generalised minimum variance self-tuning controller for nonlinear discrete-time systems. IEE Proc. Control Theory Appl..

[6] Billings S.A., Zhu Q.M. (1994). A structure detection algorithm for nonlinear dynamic rational models. Int. J. Control.

[7] Zhu Q.M., Billings S.A. (1991). Recursive parameter estimation for nonlinear rational models. J. Syst. Eng..

[8] Zhu Q.M., Billings S.A. (1993). Parameter estimation for stochastic nonlinear rational models. Int. J. Control.

[9] Aguirre L.A., Barbosa B.H.G., Braga A.P. (2010). Prediction and simulation errors in parameter estimation for nonlinear systems. Mech. Syst. Signal Process..

[10] Huo M., Duan H., Luo D., Wang Y. Parameter Estimation for a VTOL UAV Using Mutant Pigeon Inspired Optimization Algorithm with Dynamic OBL Strategy. Proceedings of the 2019 IEEE 15th International Conference on Control and Automation (ICCA).

[11] Zhu Q.M., Yu D., Zhao D. (2016). An Enhanced Linear Kalman Filter (EnLKF) algorithm for parameter estimation of nonlinear rational models. Int. J. Syst. Sci..

[12] Türksen Ö., Babacan E.K., Erçetin S. (2016). Parameter Estimation of Nonlinear Response Surface Models by Using Genetic Algorithm and Unscented Kalman Filter. Chaos, Complexity and Leadership 2014.

[13] Billings S.A., Mao K.Z. (1998). Structure detection for nonlinear rational models using genetic algorithms. Int. J. Syst. Sci..

[14] Plakias S., Boutalis Y.S. (2019). Lyapunov Theory Based Fusion Neural Networks for the Identification of Dynamic Nonlinear Systems. Int. J. Neural Syst..

[15] Kumar R., Srivastava S., Gupta J.R.P., Mohindru A. (2018). Diagonal recurrent neural network based identification of nonlinear dynamical systems with lyapunov stability based adaptive learning rates. Neurocomputing.

[16] Chen S., Liu Y. (2019). Robust Distributed Parameter Estimation of Nonlinear Systems with Missing Data over Networks. IEEE Trans. Aerosp. Electron. Syst..

[17] Zhu Q.M. (2005). An implicit least squares algorithm for nonlinear rational model parameter estimation. Appl. Math. Model..

[18] Zhu Q.M. (2003). A back propagation algorithm to estimate the parameters of non-linear dynamic rational models. Appl. Math. Model..

[19] Zhu Q.M., Wang Y., Zhao D., Li S., Billings S.A. (2015). Review of rational (total) nonlinear dynamic system modelling, identification, and control. Int. J. Syst. Sci..

[20] Leung H., Haykin S. (1993). Rational function neural network. Neural Comput..

[21] Jain R., Narasimhan S., Bhatt N.P. (2019). A priori parameter identifiability in models with non-rational functions. Automatica.

[22] Kambhampati C., Mason J.D., Warwick K. (2000). A stable one-step-ahead predictive control of nonlinear systems. Automatica.

[23] Kumar R., Srivastava S., Gupta J.R.P. (2017). Lyapunov stability-based control and identification of nonlinear dynamical systems using adaptive dynamic programming. Soft Comput..

[24] Ge H.W., Du W.L., Qian F., Liang Y.C. (2009). Identification and control of nonlinear systems by a time-delay recurrent neural network. Neurocomputing.

[25] Verdière N., Zhu S., Denis-Vidal L. (2018). A distribution input–output polynomial approach for estimating parameters in nonlinear models. Application to a chikungunya model. J. Comput. Appl. Math..

[26] Li F., Jia L. (2019). Parameter estimation of hammerstein-wiener nonlinear system with noise using special test signals. Neurocomputing.

[27] Chen C.Y., Gui W.H., Guan Z.H., Wang R.L., Zhou S.W. (2017). Adaptive neural control for a class of stochastic nonlinear systems with unknown parameters, unknown nonlinear functions and stochastic disturbances. Neurocomputing.

